# Initiation of Antipsychotics During the First Year After First‐Episode Psychosis: A Population‐Based Study

**DOI:** 10.1111/acps.13776

**Published:** 2024-11-29

**Authors:** I. Odsbu, A. Hamina, V. Hjellvik, M. Handal, M. Haram, M. Tesli, A. Tanskanen, H. Taipale

**Affiliations:** ^1^ Department of Chronic Diseases The Norwegian Institute of Public Health Oslo Norway; ^2^ Niuvanniemi Hospital Kuopio Finland; ^3^ Institute of Clinical Medicine University of Oslo Oslo Norway; ^4^ Division of Mental Health and Addiction Oslo University Hospital Oslo Norway; ^5^ Department of Mental Health and Suicide The Norwegian Institute of Public Health Oslo Norway; ^6^ Department of Psychiatry Østfold Hospital Grålum Norway; ^7^ Department of Clinical Neuroscience, Division of Insurance Medicine Karolinska Institutet Stockholm Sweden; ^8^ School of Pharmacy University of Eastern Finland Kuopio Finland

**Keywords:** antipsychotics, drug utilization, polypharmacy, psychosis, registry‐linkage

## Abstract

**Background:**

Antipsychotics are recommended after first‐episode psychosis. Knowledge on the current use patterns in real‐world settings is thus important to inform clinical practice. We aimed to describe antipsychotic initiation during 1 year after first‐episode psychosis and its associated factors.

**Methods:**

Population‐based cohort study using linked nationwide health and population registers from Norway. The study population comprised 8052 persons aged 16–45 years with first‐episode psychosis diagnosed in secondary care (ICD‐10 F20, F22–F29) in the period 2011–2019. Initiation of antipsychotic use was defined as being dispensed antipsychotics (ATC N05A, excl. lithium) at least once from −90 to +365 days from secondary care diagnosis of first‐episode psychosis. Antipsychotic polypharmacy during follow‐up was defined as having at least 90 days with overlapping drug use periods modeled using the Prescriptions to Drug Use Periods method. Adjusted risk ratios (aRRs) with 95% confidence intervals (CIs) for the association between socioeconomic and clinical factors and initiation of antipsychotic use were calculated using modified Poisson regression.

**Results:**

In total, 4413 persons (54.8%) initiated antipsychotic use after first‐episode psychosis with proportions ranging from 45.5% in 2012 to 62.1% in 2019. Oral formulations of olanzapine (34.9%), quetiapine (21.2%), and aripiprazole (11.6%) were most common at initiation, whereas long‐acting injectables (LAIs) and clozapine were rarely used. Among the initiators, 13.8% started a polypharmacy period lasting more than 90 days. Factors associated with antipsychotic initiation were lower age (aRR 1.14, 95% CI 1.08–1.21; 26–35 years vs. 36–45 years), higher education (1.11, 1.05–1.18), being employed (1.04, 1.00–1.09), being hospitalized (1.13, 1.09–1.18), being diagnosed late in the study period (1.16, 1.11–1.22; 2017–2019 vs. 2011–2013), or with previously diagnosed bipolar disorder, depression, or anxiety disorders.

**Conclusions:**

The antipsychotic use pattern is largely within the current clinical guideline. Primary non‐compliance and disease severity may explain the socioeconomic and clinical differences related to initiation of antipsychotic use.

**Summary**

- Significant Outcomes
  ○ This large‐scale registry‐based study shows that among patients aged 16–45 years with first‐episode psychosis, approximately 55% initiated antipsychotics within a year. Of those, most received monotherapy with second‐generation oral antipsychotics, which is in line with clinical guidelines.
  ○ Olanzapine was the most frequently initiated antipsychotic despite more recent international guidelines not recommending olanzapine as first‐line treatment for first‐episode psychosis.
  ○ Albeit not recommended in national clinical guidelines, antipsychotic polypharmacy during 1‐year follow‐up was evident among 14% of the antipsychotic initiators.
- Limitations
  ○ We did not have information on antipsychotics administered in inpatient care or in outpatient specialist clinics as this information is not registered in the prescription register. Lack of such information may have led to an underestimation of the proportion of antipsychotic initiators.
  ○ We did not have information about disease severity, merely ICD‐10 diagnostic codes.

## 1 Introduction

The lifetime prevalence of non‐affective psychotic disorders is 1.0%–3.5% [1, 2]. Persons experiencing a first‐episode psychosis can recover or suffer from a more chronic illness course characterized by multiple relapses [3, 4]. It is well established that antipsychotic drugs are efficient in reducing symptoms of acute psychosis and as maintenance treatment to prevent relapse in patients with schizophrenia [5, 6]. In fact, a short duration of treatment initiation may improve long‐term prognosis [7]. Consequently, international clinical guidelines and the national Norwegian guideline recommend treatment with antipsychotic drugs after first‐episode non‐affective psychosis [8, 9].

An understanding of the antipsychotic use pattern after first‐episode non‐affective psychosis is important to inform clinical practice and improve patient outcomes. To date, no studies utilizing national‐level data with long‐term follow‐up have been conducted in Norway. Additionally, only a few studies have focused on selected populations without specifically studying first‐episode psychosis [10, 11, 12]. Also, in alignment with perspectives from mental health user organizations, the Norwegian government imposed regional health trusts to provide medication‐free treatment alternatives for patients admitted to psychiatric care from 2015 and onwards [13, 14]. Whether the availability of medication‐free treatment alternatives has led to a decrease in the use of antipsychotics among patients with first‐episode psychosis is not known.

In this study, we aimed to describe the initiation of antipsychotics during the first year after first‐episode non‐affective psychosis on a national level. We investigated types and frequencies of antipsychotic drugs initiated and trends over time in the period 2011–2019. Furthermore, we investigated socioeconomic factors and type of psychosis diagnosis associated with initiation of antipsychotic use compared to no initiation. Among the antipsychotic initiators, we compared the characteristics of those receiving monotherapy with those receiving antipsychotic polypharmacy during follow‐up.

## 2 Materials and Methods

### 2.1 Data Sources, Study Population and Study Design

This was a population‐based cohort study including all persons aged 16–45 years with a first‐episode psychosis (ICD‐10 F20, F22–F29) in the period 2011–2019 (*N* = 8052). The data sources, study population, and study design are further described in Supplementary Methods, Figure S2, and Table S1.

### 2.2 Exposure

Antipsychotics were defined as all drugs in the ATC group N05A, excluding lithium (N05AN01). We further categorized antipsychotics into oral and long‐acting injectable antipsychotics (LAIs). Antipsychotic use was assessed from −90 days before admission (i.e., cohort entry) to 365 days after discharge from a first‐episode psychosis for persons diagnosed in inpatient care. For persons diagnosed in outpatient specialist care, the drug use observation window was −90 to +365 days for corresponding visit to outpatient specialist care (i.e., cohort entry) (Figure S2).

### 2.3 Antipsychotic Polypharmacy

We have studied antipsychotic polypharmacy in two ways: at initiation and during follow‐up. For the analysis of the initial antipsychotic used, antipsychotic polypharmacy at initiation was defined as being dispensed two or more antipsychotics on the same day in the drug use observation window. Here, also initiation with oral and LAI formulations of the same drug was categorized as antipsychotic polypharmacy. For the analysis of antipsychotic polypharmacy during follow‐up, antipsychotic polypharmacy was defined as continuous use of two or more antipsychotics for more than 90 days. Initiation of such a polypharmacy period was required to happen within the drug use observation window (Figure S2). Calculation of drug use periods is described in Section 2.5.

### 2.4 Follow‐Up Visit in Psychiatric Outpatient Care

As some individuals could receive antipsychotics in psychiatric outpatient care only (i.e., not dispensed through pharmacies), and this use is not captured by the NorPD, we calculated the proportion (%) of non‐initiators with at least one visit to psychiatric outpatient care during follow‐up to assess how many could potentially be treated with antipsychotics in psychiatric outpatient care.

### 2.5 Drug Use Modeling and Statistical Analyses

Drug use periods were constructed for the analysis of antipsychotic polypharmacy during follow‐up and were derived from prescription drug purchases using the Prescriptions to Drug Use Periods (PRE2DUP) method as previously described [15]. The PRE2DUP method is based on the calculation of sliding averages of defined daily dosages, the amounts of drugs purchased, and individual drug use patterns. Hospital stays and medicine stockpiling are also incorporated into the model. Taking all these parameters into account, the PRE2DUP method constructs treatment episodes representing periods where an individual most likely is exposed to a specific drug substance. Validation based on expert opinion indicates that the method creates drug use periods with a relatively high correctness [15]. If an individual used two different antipsychotics and the calculated drug use periods overlapped with at least 90 days, it was considered as antipsychotic polypharmacy (Section 2.3).

Descriptive statistics are presented as proportions with 95% confidence intervals (CIs), mean with standard deviation, and median with interquartile range. Trends in proportions of specific antipsychotic drugs initiated were calculated for the period 2011–2019 using linear regression. We performed modified Poisson regression with robust error variance estimation to assess the association between socioeconomic and clinical factors and initiation of antipsychotic use after first‐episode psychosis [16]. A detailed description of the covariates is provided in Supplementary Methods. In the adjusted model, all socioeconomic and clinical covariates were included, except previous use of anxiolytics, hypnotics or antidepressants as these variables were highly correlated. The results are reported as risk ratios (RRs) with 95% CIs.

Statistical analyses were performed with Stata version 17 (Stata Corp, TX, USA).

## 3 Results

### 3.1 Characteristics of the Study Population

The mean age at cohort entry was 29.7 years and there was a higher proportion of males (61.1%) (Table 1). Most were diagnosed with a first‐episode psychosis in psychiatric outpatient clinics (61.8%). For those hospitalized at the first episode, the median duration of the hospital stay was 6 days (IQR 1–20). The most frequent first diagnoses recorded were acute and transient psychotic disorder (F23; 26.1%), unspecified non‐organic psychosis (F29; 25.7%), and schizophrenia (F20; 21.4%). The highest proportion of the study population was diagnosed first time in the period 2017–2019 (42.4% vs. 26.4% in the period 2011–2013).

**TABLE 1:** Baseline characteristics of the study population at cohort entry.

	*N* = 8052
Age, mean (SD)	29.7 (8.3)
Age categories, *n* (%)	
16–25	2920 (36.3)
26–35	2868 (35.6)
36–45	2264 (28.1)
Males, *n* (%)	4921 (61.1)
Education, *n* (%)	
Lower secondary school or less	4262 (52.9)
Upper secondary school	2019 (25.1)
Tertiary education	1349 (16.8)
No data on education	422 (5.2)
Employed during the year before cohort entry, *n* (%)	2520 (31.3)
Disability pension during the year before cohort entry, *n* (%)	307 (3.8)
Living alone, *n* (%)	2711 (33.7)
Source of first diagnosis, *n* (%)	
Inpatient care	3080 (38.3)
Outpatient specialist care	4972 (61.8)
Length of stay for inpatients (days), median (IQR)	6 (1–20)
Type of first diagnoses (ICD‐10), *n* (%)	
Acute and transient psychotic disorder (F23)	2104 (26.1)
Unspecified nonorganic psychosis (F29)	2071 (25.7)
Schizophrenia (F20)	1719 (21.4)
Persistent delusional disorder (F22)	1504 (18.7)
Schizoaffective disorder (F25)	279 (3.5)
Other nonorganic psychotic disorder (F28)	224 (2.8)
Unspecified^a^	151 (1.9)
Calendar year of cohort entry, *n* (%)	
2011–2013	2126 (26.4)
2014–2016	2516 (31.3)
2017–2019	3410 (42.4)
Outpatient specialist care contact during first year of follow‐up, *n* (%)	
No	3933 (48.9)
Yes	4119 (51.2)
Previously diagnosed mental disorders, *n* (%)	
Substance use disorder	1992 (24.7)
Bipolar disorder	337 (4.2)
Depression	1658 (20.6)
Anxiety disorder	1217 (15.1)
Stress‐related disorder	1126 (14.0)
Personality disorder	677 (8.4)
ADHD	565 (7.0)
Other medication use during 90 days before cohort entry	
Anxiolytic use	2383 (29.6)
Hypnotic use	2819 (35.0)
Antidepressant use	2788 (34.6)

Abbreviations: ICD‐10 = International Classification of Diseases, 10th revision; IQR = interquartile range; SD = standard deviation.

^a^ Not specified on ICD‐10 three‐character level.

### 3.2 First Antipsychotic Initiated

**FIGURE 1:**
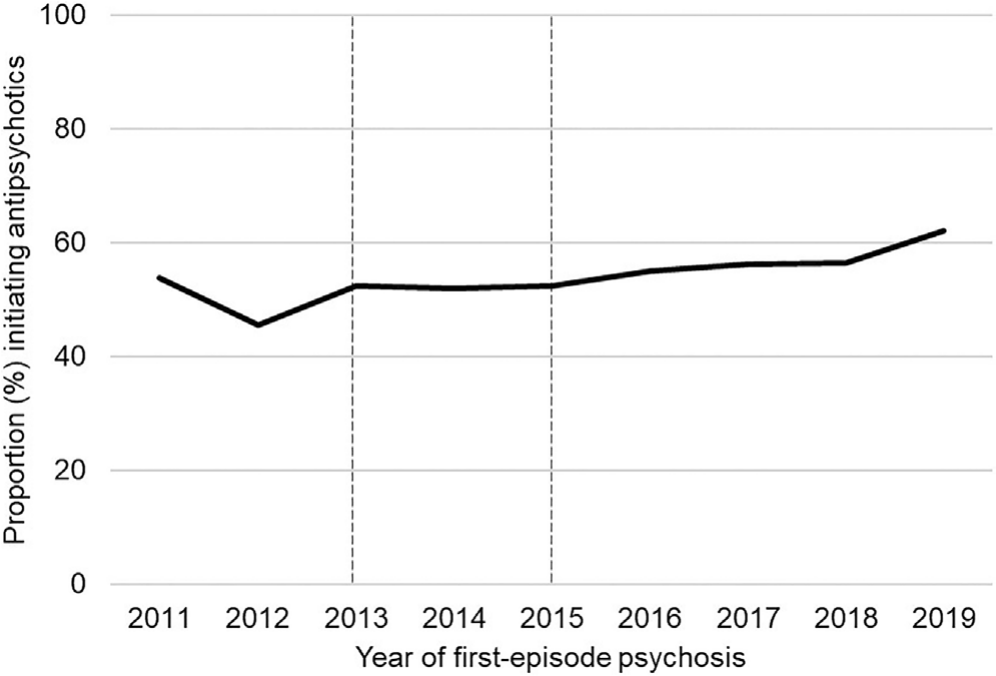
Proportion (%) initiating antipsychotic use within the period from 3 months before to 1 year after first‐episode psychosis. The denominator is the number of individuals with a first‐episode psychosis in the respective years. The dashed lines mark the implementation of the current treatment guideline (in 2013) and the launch of medication‐free treatment alternatives for patients admitted to psychiatric care (in 2015).

**FIGURE 2:**
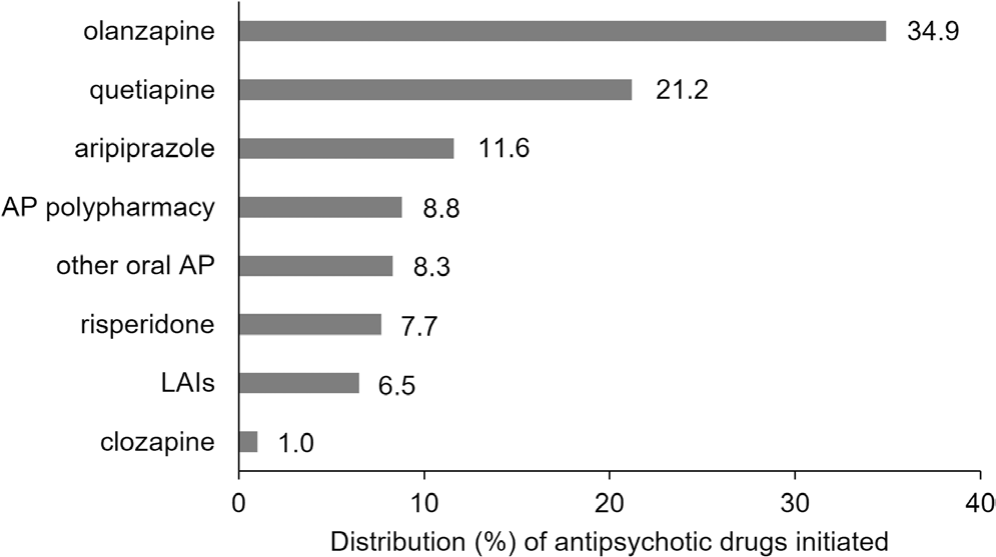
Distribution (%) of antipsychotic drug initiated within the first year after first‐episode psychosis. The denominator is number of individuals initiating antipsychotics during 2011–2019 (*N* = 4413). Specific drug substances refer to oral antipsychotics and all long‐acting injectables are categorized as “LAIs.” AP polypharmacy refers to situations when two or more antipsychotics were dispensed on the same day. Also, initiation with oral and LAI formulation of the same drug substance is categorized as AP polypharmacy.

Overall, there were 4413 persons (54.8%) initiating antipsychotic use within the period from 3 months before to 1 year after first‐episode psychosis with proportions ranging from 45.5% in 2012 to 62.1% in 2019 (Figure 1). The most common antipsychotics used at initiation were oral formulations of olanzapine (34.9%), quetiapine (21.2%), and aripiprazole (11.6%) (Figure 2). LAIs were initiated among 6.5% of the cases. Clozapine was rarely initiated (1.0%). The proportion of antipsychotic polypharmacy at initiation was 8.8% (*N* = 389) and among those, almost all (95.5%) started with two drugs. The three most common combinations were olanzapine and quetiapine (*N* = 90), aripiprazole and quetiapine (*N* = 50), and risperidone and quetiapine (*N* = 36). Filling prescriptions of both oral and depot formulations of the same drug substance was rare (*N* = 6).

### 3.3 Trends in Antipsychotic Drug Use at Initiation

**FIGURE 3:**
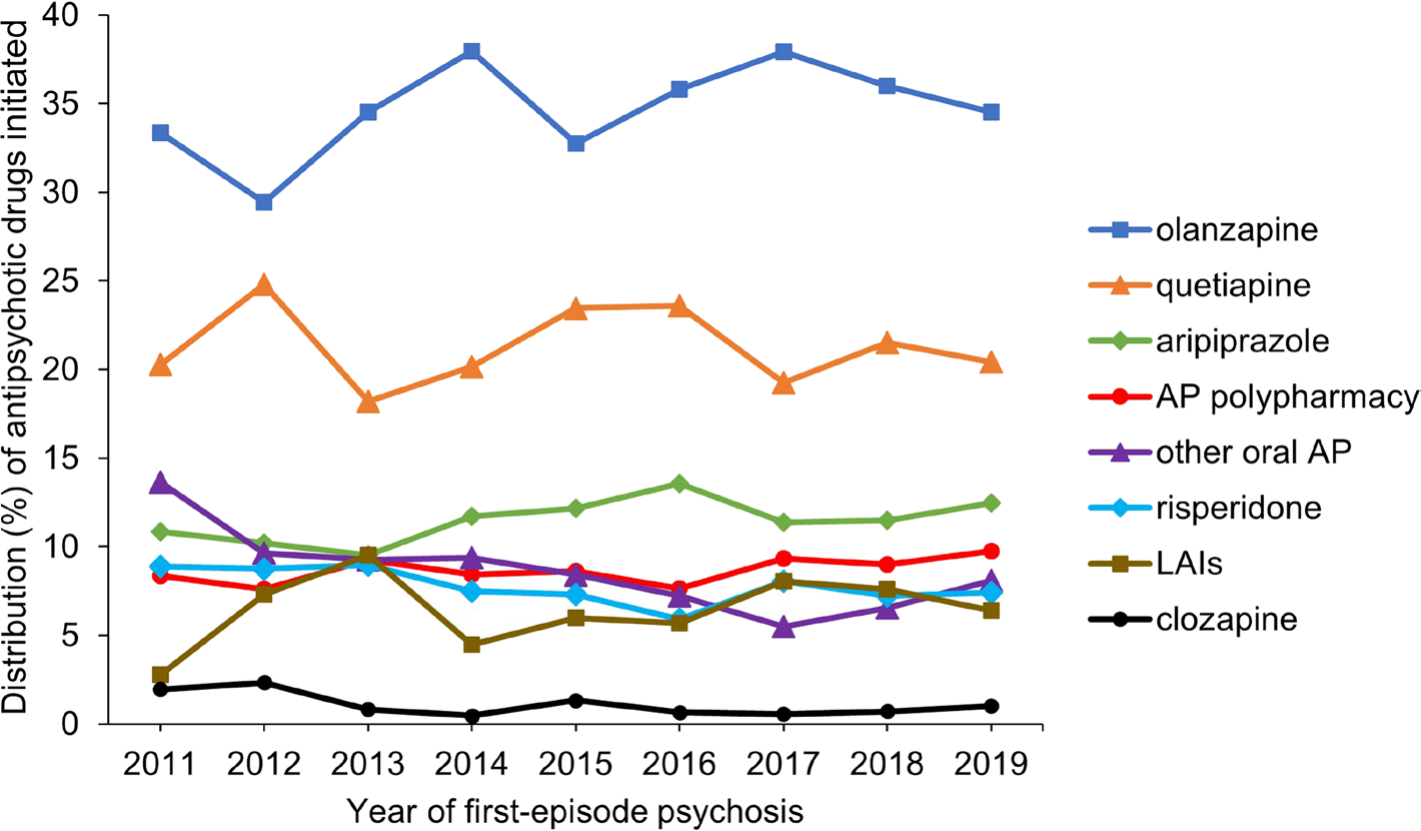
Distribution (%) of antipsychotic drug initiated within the first year after first‐episode psychosis according to cohort entry year in the period 2011–2019. The denominator is the number of individuals initiating antipsychotics in the respective years. Specific drug substances refer to oral antipsychotics and all long‐acting injectables are categorized as “LAIs.” AP polypharmacy refers to situations when two or more antipsychotics were dispensed on the same day. Also, initiation with oral and LAI formulation of the same drug substance is categorized as AP polypharmacy.

Over the period 2011–2019, initiation with clozapine and other oral antipsychotics (mainly first‐generation antipsychotics) showed statistically significant decreasing trends (Figure 3 and Table S3). No significant changes were observed for the other antipsychotic drugs. Olanzapine remained the most frequently initiated antipsychotic drug throughout the period (34.5% in 2019), followed by quetiapine (20.4% in 2019) and aripiprazole (12.4% in 2019). The pattern of antipsychotic polypharmacy at initiation was quite stable, whereas the use of LAIs showed larger variations, particularly in the first half of the study period.

### 3.4 Factors Associated With Initiation of Antipsychotic Use

Initiation of antipsychotic use was associated with lower age (adjusted RR 1.14, 95% CI 1.08–1.21 for the age group 26–35 years compared to the oldest age group 36–45 years), higher education (1.11, 1.05–1.18), being employed (1.04, 1.00–1.09), and being hospitalized (inpatient care) (1.13, 1.09–1.18) (Table 2). There was no difference between males and females. Compared to those being diagnosed with acute and transient psychotic disorder (F23), those being diagnosed with persistent delusional disorder (F22), schizophrenia (F20), unspecified nonorganic psychosis (F29), or other (F24/F25/F28/unspecified) diagnoses were less likely to initiate antipsychotics. Individuals diagnosed late in the study period had a higher risk of using antipsychotics compared to those being diagnosed early in the study period (1.16, 1.11–1.22 for 2017–2019 vs. 2011–2013). Other previously diagnosed mental disorders associated with initiation of antipsychotics were bipolar disorder (1.22, 1.13–1.32), depression (1.08, 1.03–1.13), and anxiety disorders (1.05, 1.00–1.11).

**TABLE 2:** Unadjusted and adjusted risk ratios (RRs) with 95% confidence intervals (CIs) for the association between sociodemographic and clinical factors and the initiation of antipsychotic use (vs. no use) at 1 year after diagnosis of first‐episode psychosis in Norway.

	No use (*N* = 3639)		Initiation of AP (*N* = 4413)		Unadjusted RR (95% CI)	Adjusted RR (95% CI)
	*n*	%	*n*	%		
Age categories						
16–25	1250	34.4	1670	37.8	1.08 (1.02–1.13)	1.09 (1.03–1.15)
26–35	1285	35.3	1583	35.9	1.12 (1.06–1.17)	1.14 (1.08–1.21)
36–45	1104	30.3	1160	26.3	ref	ref
Sex						
Males	2256	62.0	2665	60.4	ref	ref
Females	1383	38.0	1748	39.6	1.03 (0.99–1.07)	1.01 (0.97–1.05)
Education						
Lower secondary education or less	1945	53.5	2317	52.5	ref	ref
Upper secondary school	914	25.1	1105	25.0	1.03 (0.96–1.06)	1.03 (0.98–1.09)
Tertiary education	557	15.3	792	18.0	1.08 (1.02–1.14)	1.11 (1.05–1.18)
No data on education	223	6.1	199	4.5	0.87 (0.78–0.96)	0.86 (0.78–0.97)
Employed during the year before cohort entry	1082	29.7	1438	33.6	1.06 (1.02–1.11)	1.04 (1.00–1.09)
Disability pension	154	4.2	153	3.5	0.91 (0.81–1.02)	0.98 (0.87–1.09)
Living alone	1296	35.6	1415	32.1	0.93 (0.89–0.97)	0.96 (0.91–1.00)
Source of first diagnosis						
Inpatient care	1251	34.4	1829	41.5	1.15 (1.10–1.19)	1.13 (1.09–1.18)
Outpatient specialist care	2388	65.5	2584	58.6	ref	ref
Type of first diagnoses (ICD‐10)						
Acute and transient psychotic disorder (F23)	817	22.5	1287	29.2	ref	ref
Persistent delusional disorder (F22)	701	19.3	803	18.2	0.87 (0.82–0.93)	0.91 (0.86–0.97)
Schizophrenia (F20)	836	23.0	883	20.0	0.84 (0.79–0.89)	0.90 (0.85–0.95)
Unspecified nonorganic psychosis (F29)	975	26.8	1096	24.8	0.87 (0.82–0.91)	0.87 (0.83–0.92)
Other/unspecified psychosis^a^	310	8.5	344	7.8	0.86 (0.79–0.93)	0.88 (0.81–0.95)
Calendar year of cohort entry						
2011–2013	1055	29.0	1071	24.3	ref	ref
2014–2016	1179	32.4	1337	30.3	1.05 (1.00–1.12)	1.05 (0.99–1.11)
2017–2019	1405	38.6	2005	45.4	1.17 (1.11–1.23)	1.16 (1.11–1.22)
Previously diagnosed mental disorders						
Substance use disorder	880	24.2	1112	25.2	1.03 (0.98–1.07)	1.03 (0.98–1.08)
Bipolar disorder	111	3.1	226	5.1	1.24 (1.14–1.34)	1.22 (1.13–1.32)
Depression	682	18.7	976	22.1	1.10 (1.05–1.15)	1.08 (1.03–1.13)
Anxiety disorder	511	14.0	706	16.0	1.07 (1.01–1.13)	1.05 (1.00–1.11)
Stress‐related disorder	477	13.1	649	14.7	1.06 (1.00–1.12)	1.02 (0.97–1.08)
Personality disorder	308	8.5	369	8.4	0.99 (0.93–1.07)	0.97 (0.90–1.05)
ADHD	231	6.4	334	7.6	1.09 (1.01–1.17)	1.05 (0.98–1.13)

Abbreviations: AP = antipsychotics; ICD‐10 = International Classification of Diseases, 10th revision.

^a^ The rest grouped together.

### 3.5 Antipsychotic Polypharmacy During Follow‐Up

The proportion of persons who initiated antipsychotic polypharmacy lasting more than 90 days during the first year was 13.8% (*N* = 607). Of those, 37.9% (*N* = 230) initiated polypharmacy already when they initiated antipsychotic use, 36.2% (*N* = 220) initiated polypharmacy during 1–90 days, 11.2% (*N* = 68) initiated during 91–180 days and 14.7% (*N* = 89) initiated more than 6 months after initiation. Among the antipsychotic initiators, those who had polypharmacy during follow‐up were more often diagnosed with schizophrenia, having previously diagnosed substance use disorder, and living alone when compared to those receiving monotherapy during follow‐up (Table 3). They were less likely to be diagnosed with acute and transient psychotic disorder (F23) when compared to those on monotherapy.

**TABLE 3:** Characteristics of antipsychotic initiators according to whether they received monotherapy only or polypharmacy (more than 90 days) during follow‐up.

	Monotherapy (*N* = 3806)	95% confidence interval	Polypharmacy (*N* = 607)	95% confidence interval
Age categories, *n* (%)				
16–25	1455 (38.2)	36.7–39.8	215 (35.4)	31.6–39.4
26–35	1358 (35.7)	34.2–37.2	225 (37.1)	33.2–41.1
36–45	993 (26.1)	24.7–27.5	167 (27.5)	24.0–31.3
Sex, *n* (%)				
Males	2290 (60.2)	58.6–61.7	375 (61.8)	57.8–65.6
Females	1516 (39.8)	38.3–41.4	232 (38.2)	34.4–42.2
Education, *n* (%)				
Lower secondary school or less	1998 (52.5)	50.9–54.1	319 (52.6)	48.5–56.6
Upper secondary school	939 (24.7)	23.3–26.1	166 (27.4)	23.9–31.1
Tertiary education	699 (18.4)	17.2–19.6	93 (15.3)	12.6–18.5
No data on education	170 (4.5)	3.8–5.2	29 (4.8)	3.3–6.9
Employed during the year before cohort entry, *n* (%)	1262 (33.2)	31.7–34.7	176 (29.0)	25.5–32.8
Disability pension, *n* (%)	124 (3.3)	2.7–3.9	29 (4.8)	3.3–6.9
Living alone, *n* (%)	1210 (31.8)	30.3–33.3	305 (50.2)	46.2–54.3
Source of first diagnosis, *n* (%)				
Inpatient care	1573 (41.3)	39.8–42.9	256 (42.2)	38.2–46.2
Outpatient specialist care	2233 (58.7)	57.1–60.2	351 (57.8)	53.8–61.8
Type of first diagnoses (ICD‐10), *n* (%)				
Acute and transient psychotic disorder (F23)	1138 (29.9)	28.5–31.4	149 (24.6)	21.2–28.2
Persistent delusional disorder (F22)	708 (18.6)	17.4–19.9	95 (15.7)	12.9–18.8
Schizophrenia (F20)	722 (19.0)	17.7–20.3	161 (26.5)	23.1–30.3
Unspecified nonorganic psychosis (F29)	953 (25.0)	23.7–26.5	143 (23.6)	20.3–27.2
Other/unspecified psychosis^a^	285 (7.5)	6.7–8.4	59 (9.7)	7.5–12.4
Calendar year of cohort entry, *n* (%)				
2011–2013	928 (24.4)	23.0–25.8	143 (23.6)	20.3–27.2
2014–2016	1152 (30.3)	28.8–31.8	185 (30.5)	26.9–34.3
2017–2019	1726 (45.4)	43.8–47.0	279 (46.0)	42.0–50.0
Previously diagnosed mental disorders, *n* (%)				
Substance use disorder	924 (24.3)	22.9–25.7	188 (31.0)	27.3–34.8
Bipolar disorder	192 (5.0)	4.4–5.8	34 (5.6)	4.0–7.8
Depression	837 (22.0)	20.7–23.4	139 (22.9)	19.7–26.5
Anxiety disorder	590 (15.5)	14.4–16.7	116 (19.1)	16.1–22.5
Stress‐related disorder	554 (14.6)	13.5–15.7	95 (15.7)	12.9–18.8
Personality disorder	309 (8.1)	7.3–9.0	60 (9.9)	7.7–12.6
ADHD	285 (7.5)	6.7–8.4	49 (8.1)	6.1–10.6

Abbreviation: ICD‐10 = International Classification of Diseases, 10th revision.

^a^ The rest grouped together.

### 3.6 Follow‐Up Visit in Psychiatric Outpatient Care During the First Year After First‐Episode Psychosis

Among those who did not initiate antipsychotic use (*N* = 3639), 42.4% had at least one visit in psychiatric outpatient care during the first year. The corresponding proportion was 58.4% among those who initiated antipsychotic use. In total, 26% (2096/8052) of the study population neither initiated antipsychotics nor visited a psychiatric outpatient clinic during the first year.

## 4 Discussion

In this population‐based study, we identified 8052 persons aged 16–45 years with a first‐episode psychosis during 2011–2019. Among them, 54.8% initiated antipsychotic use in the period from 3 months before to 1 year after diagnosis. Monotherapy was most common at initiation. Those initiating antipsychotics were of younger age, had higher education, were more likely employed the previous year, more often diagnosed in inpatient care or late in the study period, and more often previously diagnosed with bipolar disorder, depression, or anxiety disorder compared to those not initiating antipsychotics. Among those with antipsychotic use, 13.8% initiated a polypharmacy period lasting for more than 90 days. Compared to those on monotherapy, they were more often diagnosed with schizophrenia at their first psychosis episode, had a previously diagnosed substance use disorder, or were living alone.

We found that just over half of the patients with first‐episode psychosis initiated antipsychotics and that one in four patients neither initiated antipsychotics nor visited a psychiatric outpatient clinic during the 1‐year follow‐up. In a recent report from a psychiatric unit in the capital area of Norway treating patients aged 17–30 years with first‐episode psychosis, it was found that 80% of the patients were treated with antipsychotics whereas 20% did not use any antipsychotic medication [17]. This could be due to long‐term follow‐up, where patients have tapered the use of antipsychotic medication. Patients admitted to this unit are patients that have not responded to treatment provided elsewhere in the last 3–6 months and/or who are not functioning well. Population‐based studies from Sweden and Finland have previously found that 80% and 58% of patients with a first hospitalization for schizophrenia filled an antipsychotic prescription within 6 months and 30 days after discharge, respectively [18, 19]. In the present study, we have used a broader definition of first‐episode psychosis and included both hospitalizations and outpatient specialist care possibly leading to the inclusion of patients with different disease severity. Also, a registration in the national patient register could be a referral diagnosis that is registered upon the first visit or a tentative diagnosis that is set during diagnostic work up which later turns out not to fulfill criteria for a psychotic disorder, for example, if someone had been under the influence of substances or pharmacological treatment that led to a psychotic episode. Hence, the lower proportion of patients filling an antipsychotic prescription in the present study could be that there are more patients not in need of antipsychotic medication. A recent scoping review on the global psychotropic prescription patterns in schizophrenia found a proportion of antipsychotic use of 67%–99% and that most received monotherapy (52%–91%) [20].

According to the Norwegian guideline for treatment of psychosis (last updated in 2013), monotherapy with any of the second‐generation antipsychotics is recommended and antipsychotic polypharmacy is advised against [8]. Treatment with clozapine is an option if monotherapy with two different second‐generation antipsychotics has failed. Overall, we found that the antipsychotic initiation pattern after first‐episode psychosis was in line with clinical recommendations as second‐generation antipsychotic monotherapy was the most common. During the study period, the use of first‐generation antipsychotics declined, and initiation of clozapine was rare. In the guideline, there is no specific advice on which second‐generation antipsychotic should be tried first, but the choice should rather be based on tolerability and side effects of the drug. We found that oral formulation of olanzapine was the most used antipsychotic drug despite its metabolic side effects and cardiac risks [21]. This is similar to population‐based studies from other Nordic countries [18, 19, 22]. Based on emerging evidence of the associated risks with olanzapine use, several international guidelines do not recommend olanzapine as the first choice, including the Swedish and Danish guidelines on treatment of psychosis [9, 23, 24, 25]. Possible explanations for the frequent use of olanzapine could be that sedation is important in the acute treatment phase and that olanzapine reduces positive symptoms effectively [26]. Olanzapine is also available as depot formulation, and it can be more convenient to switch to depot formulation of the same drug substance instead of switching to an antipsychotic with a better side effect profile, such as aripiprazole (also available as depot formulation). However, obligatory monitoring is mandatory after each olanzapine injection as opposed to other antipsychotics available as depot formulation. Investigating switching between drug substances during follow‐up was outside the scope of this study.

We identified socioeconomic and clinical factors associated with initiation of antipsychotics that could reflect issues of primary non‐adherence and disease severity. A previous Finnish study found that 31.7% of patients with schizophrenia prescribed antipsychotics did not fill the prescription within the following year [27]. Contrary to our findings, young age and female sex were both related to primary non‐adherence in Finland. Our finding that patients with higher education were more likely to initiate antipsychotics is in line with previous studies showing an association between low education and poor medication adherence [28, 29]. Employment the previous year before first‐episode psychosis could indicate better functioning and was found to be associated with antipsychotic initiation in our study. Patients diagnosed with acute and transient psychotic disorder were more likely to initiate antipsychotics than patients receiving other diagnoses such as schizophrenia. Possible explanations for this could be that for the most severe conditions such as schizophrenia, patients may be more likely to exhibit primary non‐compliance, or that antipsychotics are administered at the expense of the hospital and thus not registered in the prescription register. We found that being hospitalized at the first psychosis episode and being diagnosed late in the study period were both associated with initiation of antipsychotic treatment. The former could possibly be explained by hospitalized patients being stabilized on medication upon discharge and continuing treatment in outpatient specialist care or primary care, and also that they more likely had a proper clinical first episode of psychosis, were more severely ill, and more in need of medication. The reason for the latter is not known, but one possible explanation for the observation that a higher proportion of the study population was diagnosed at the end of the study period could be changes in diagnostic practice in recent years. In 2017, there was a major change in the Mental Health Act requiring that the patient's competence to consent must be assessed before deciding which treatment could be given further. The main aim was to reduce coercive treatment, but a recent report from the Norwegian Ministry of Health and Care Services noted that coercive admissions in fact increased [30]. The amendment could have led to an overall change in diagnostic practice, with more patients with unclear presentation of symptoms and low functioning being assessed as having “possible psychosis.” However, this particular association is yet to be investigated.

Although use of two or more antipsychotics is advised against, 8.8% of the antipsychotic initiators were dispensed two or more antipsychotics at initiation. Quetiapine in combination with olanzapine, aripiprazole, or risperidone was most common. As quetiapine is frequently prescribed for sleep disorders and anxiety in lower dosages, this prescribing pattern could reflect add‐on therapy to treat these symptoms rather than antipsychotic polypharmacy for treatment of psychotic symptoms. It is important to note that filled prescriptions of oral formulations and LAIs of the same drug substance at initiation were also considered to be antipsychotic polypharmacy. Such a combination would likely reflect proper clinical practice where a shorter period with both tablets and LAIs is often necessary to obtain the correct serum concentration during the titration phase. However, there were very few of the polypharmacy initiators that had such a combination. It is possible that titration phases are not captured in the prescription register since initiation often occurs in inpatient or outpatient specialist care.

We found that 13.8% of the antipsychotic users initiated a polypharmacy period lasting for more than 90 days within the first year of follow‐up and that among these patients, one‐third started such a polypharmacy period already at initiation of antipsychotic treatment. An overlapping period of 90 days likely reflects concomitant use and not a period of switching from oral to depot formulation or between different antipsychotics. The proportion is lower than reported in previous studies from Norway (from 20% to 35.6%) [10, 11, 12], but closer to the recent report from a psychiatric unit where 90% of the antipsychotic users received monotherapy [17]. The previous Norwegian studies included patients with schizophrenia admitted to a maximum‐security psychiatric unit in mid‐Norway in the period 1987–2000 [12], admitted to acute psychiatric wards during a 3‐month period in 2005 [11], and from psychiatric hospitals in the capital area from 2003 to 2010 [10], and thus do not represent a national sample. Also, they were all based on data collected before 2011, and do not describe the current situation. The proportion with antipsychotic polypharmacy during follow‐up was also lower compared to national samples of patients with first‐episode schizophrenia in Sweden (26.8%) and Finland (22.7%) [22]. In the present study, we did however find that patients receiving polypharmacy were more often diagnosed with schizophrenia at their first psychosis episode compared to those on monotherapy. Bolstad et al. found that previous admissions to hospital and more severe symptoms were related to treatment with two or more antipsychotics [10], while Kroken et al. found that younger age, inpatient treatment in the previous 12 months, or a comorbid diagnosis of personality disorder or mental retardation were associated with polypharmacy [11]. In our study, we did not have information about symptom severity, and we also investigated patients with a first record of psychosis diagnosis in inpatient or outpatient specialist care, that is, with no previous treatment. Taken together, our results suggest that antipsychotic polypharmacy is less frequent among patients with first‐episode psychosis compared to cases with a longer history of psychosis episodes.

The implementation of medication‐free treatment alternatives in 2015 does not appear to have led to a reduction in the use of antipsychotics after first‐episode psychosis. Rather, we found that the proportion of patients initiating antipsychotics was slightly higher after 2015 as compared to before. A qualitative study exploring patients' reasons for seeking medication‐free treatment revealed that all recruited participants had many years of severe mental illness and many years of antipsychotics use [13]. Hence, it seems that patients with first‐episode psychosis do not represent most patients seeking such care.

A major strength of this study was utilizing linked data from the national health and population registers which allowed for studying unselected populations. We identified all persons with a first episode of non‐affective psychosis using a 3‐year wash‐out period. As we did not have information about substance‐induced psychosis (F1x.5), we cannot rule out that some people had a previously recorded diagnosis of substance‐use‐induced psychosis (i.e., before cohort entry). We did, however, exclude persons receiving antipsychotics in the period −450 to −90 days before first‐episode psychosis, meaning that those with substance‐induced psychosis that received treatment with antipsychotics in that period were not included in the study. Since first‐episode psychosis was defined as a first treatment contact in specialist health care, we may not have captured individuals early in the course of a psychotic illness [31]. Also, the requirement of only one recorded diagnosis may have led to the inclusion of individuals without a proper clinical diagnosis. We did not have information on diagnoses from primary care. As many patients with psychotic disorders are followed up in primary care and many do not receive antipsychotics in longer periods of time, it could be that some of the included individuals are not cases with first‐episode psychosis. Information on dispensed antipsychotics was used as proxy for antipsychotic use. Although we did eliminate primary non‐compliance, we do not know whether the patients used the medication. Adherence to antipsychotic medication is generally low among people with schizophrenia, and lower for oral formulations compared to LAIs [19, 32, 33, 34]. In Norway, it is not uncommon that LAIs are administered in outpatient specialist care and this information is not captured in the NorPD. In addition, oral antipsychotic drugs provided with coercive treatment are administered without costs for the patient in outpatient specialist care, and not captured in NorPD. Indeed, comparison of sales data from the wholesaler‐based drug statistics in Norway with data on dispensed drugs from the NorPD shows that approximately 60% of the total amount of LAIs sold are captured in the NorPD, whereas more than 80% of total sales are captured for oral formulations (amount measured as number of DDDs; data not shown) [35]. More than 40% of the non‐initiators had at least one visit in outpatient specialist care during follow‐up showing that the proportion of initiators reported in this study is likely underestimated as these could have been administered antipsychotics in outpatient specialist care. Finally, the exclusion of individuals dying during the 1‐year follow‐up period may have biased the study population towards healthier patients.

In conclusion, the antipsychotic use pattern after first‐episode psychosis is within the current clinical guideline and has remained relatively stable during 2011–2019. However, the predominant use of olanzapine at initiation is not in line with recommendations from more recent international guidelines. The implementation of medication‐free treatment alternatives has not led to a reduction in antipsychotic use after first‐episode psychosis. On the contrary, we found that a higher proportion of patients initiated antipsychotics in recent years. There are socioeconomic and clinical differences between those initiating antipsychotics and those who do not, likely reflecting differences in primary non‐adherence and disease severity. Although concurrent use of two or more antipsychotics is advised against, it is evident from our data that a smaller proportion of patients received such treatment during the following year.

## Author Contributions

I.O. drafted the manuscript. I.O., A.H., Marte Handal, A.T., H.T. conceived the idea. I.O. and Marte Handal contributed to data acquisition. V.H. performed the data management. A.T. and H.T. conducted the drug use modeling and statistical analyses. All authors interpreted the results and approved the final version of the manuscript.

## Ethics Statement

Ethical approval was secured from the Regional Ethical Research Board in Norway (2017/2225/REK sør‐øst C).

## Conflicts of Interest

H.T. and A.T. have participated in research projects funded by Janssen. H.T. reports personal fees from Gedeon Richter, Janssen, Lundbeck, and Otsuka. M.T. has received speaker's honorarium from Lundbeck and Otsuka. Marit Haram received speaker's honorarium from Lundbeck. Other authors declare no conflicts of interest.

### Peer Review

The peer review history for this article is available at https://www.webofscience.com/api/gateway/wos/peer‐review/10.1111/acps.13776.

## Supporting information


Data S1.

## Data Availability

The register‐based cohort is based on individual‐level data from the national health and population registers. The authors are not allowed, by law, to publicly share this data. Therefore, the authors cannot make this data fully available to the public. The authors may share statistical code.
