# Supplementary material for: Initiation of Antipsychotics During the First Year After First‐Episode Psychosis: A Population‐Based Study
Source: Acta Psychiatr Scand. 2024 Nov 29;151(4):537–47. doi: 10.1111/acps.13776 (PMC11884914; doi:10.1111/acps.13776)
Supplement: Supplementary file 1 — Data S1. [file ACPS-151-537-s001.docx]

**SUPPLEMENTARY MATERIAL**

Odsbu I, Hamina A, Hjellvik V et al. Initiation of antipsychotics during the first year after first-episode psychosis: a population-based study

**Supplementary methods**

1.1 Description of data sources

1.2 Description of study design and study population

1.3 Description of covariates

**Figure S1.** Flowchart for selection of the study population.

**Figure S2.** Illustration of study design and assessment periods.

**Table S1.** ICD-10 codes for psychosis-spectrum disorders included in the study.

**Table S2.** Definition of covariates and assessment periods.

**Table S3.** Distribution (%) of antipsychotic drugs initiated within the first year after first-episode non-affective psychosis according to cohort entry year during 2011-2019. The denominator is number of individuals initiating antipsychotics the respective years.

**Supplementary methods**

**1.1 Description of data sources**

Data from the Norwegian Patient Registry (NPR; period 2008-2019), the Norwegian Prescription Database (NorPD; period October 2009-2020), the Cause of Death Registry (CDR; period 2011-2020), and Statistics Norway (period 2010-2020) were linked via each person’s personal identification number (PIN). We retrieved data on hospitalizations and outpatient specialist care visits from the NPR^1^ where diagnoses are recorded according to the International Classification of Diseases 10th revision (ICD-10). The NorPD includes data on dispensed drugs from all pharmacies in Norway, classified according to the Anatomical Therapeutic Chemical (ATC) classification system^2^. These data include information on dispensation date and detailed information about the purchase such as package size and strength^3^. Data on drugs administered in hospitals or outpatient specialist care are not captured. Information on date of death was retrieved from the CDR and information on date of emigration and sociodemographic variables were retrieved from Statistics Norway.

**References:**

^1^ Bakken IJ, Ariansen AMS, Knudsen GP, Johansen KI, Vollset SE. The Norwegian Patient Registry and the Norwegian Registry for Primary Health Care: Research potential of two nationwide health-care registries. Scand J Public Health. Feb 2020;48(1):49-55. doi:10.1177/1403494819859737

^2^ WHO. ATC classification index with DDDs 2023. WHO collaborating centre for drug statistics methodology, Oslo, Norway. Available from: https://www.whocc.no/atc_ddd_index_and_guidelines/atc_ddd_index/ [accessed 05.07.2023].

^3^ Furu K, Wettermark B, Andersen M, Martikainen JE, Almarsdottir AB, Sørensen HT. The Nordic countries as a cohort for pharmacoepidemiological research. Basic Clin Pharmacol Toxicol. Feb 2010;106(2):86-94. doi:10.1111/j.1742-7843.2009.00494.x

**1.2. Description of study design and study population**

We identified all persons with at least one registration of psychosis-spectrum diagnosis in the period 1 January 2008 to 31 December 2019 (ICD-10 F20, F22-F29) (Figure S1, Table S1). To identify persons with a first-episode psychosis, we excluded everyone with a diagnosis before 1 January 2011 (3-year wash-out). For those first diagnosed in outpatient specialist care, the date of the visit was considered as cohort entry date (Day 0), and for those first diagnosed in inpatient care, the admission date was considered as the cohort entry date. Among the incident cases, we excluded everyone with dispensed antipsychotics in the period -450 to -90 days before cohort entry (except for dispensations of low-dose quetiapine, i.e., tablet strengths of 25 or 50 mg), those with their first hospital stay lasting longer than 180 days, those who emigrated or died within 365 days of follow-up, and those aged below 16 or above 45 years at cohort entry. The final study population consisted of 8052 persons aged 16-45 years with a first-episode psychosis in the period 2011-2019. The study design and assessment periods are illustrated in Figure S2.

**1.3 Description of covariates**

Sociodemographic covariates were highest level of achieved education, employment status, receiving disability pension, and living alone, all assessed one year before cohort entry. Clinical covariates were previously diagnosed mental disorders other than non-affective psychosis defined as at least one registered diagnosis in the period of three years before cohort entry. Other drugs studied were ATC groups N05B (anxiolytics), N05C (hypnotics and sedatives), and N06A (antidepressants), and use of these drugs was defined as at least one filled prescription in the period of three months before cohort entry. A detailed description of covariate definitions and assessment periods is provided in Table S2.

**
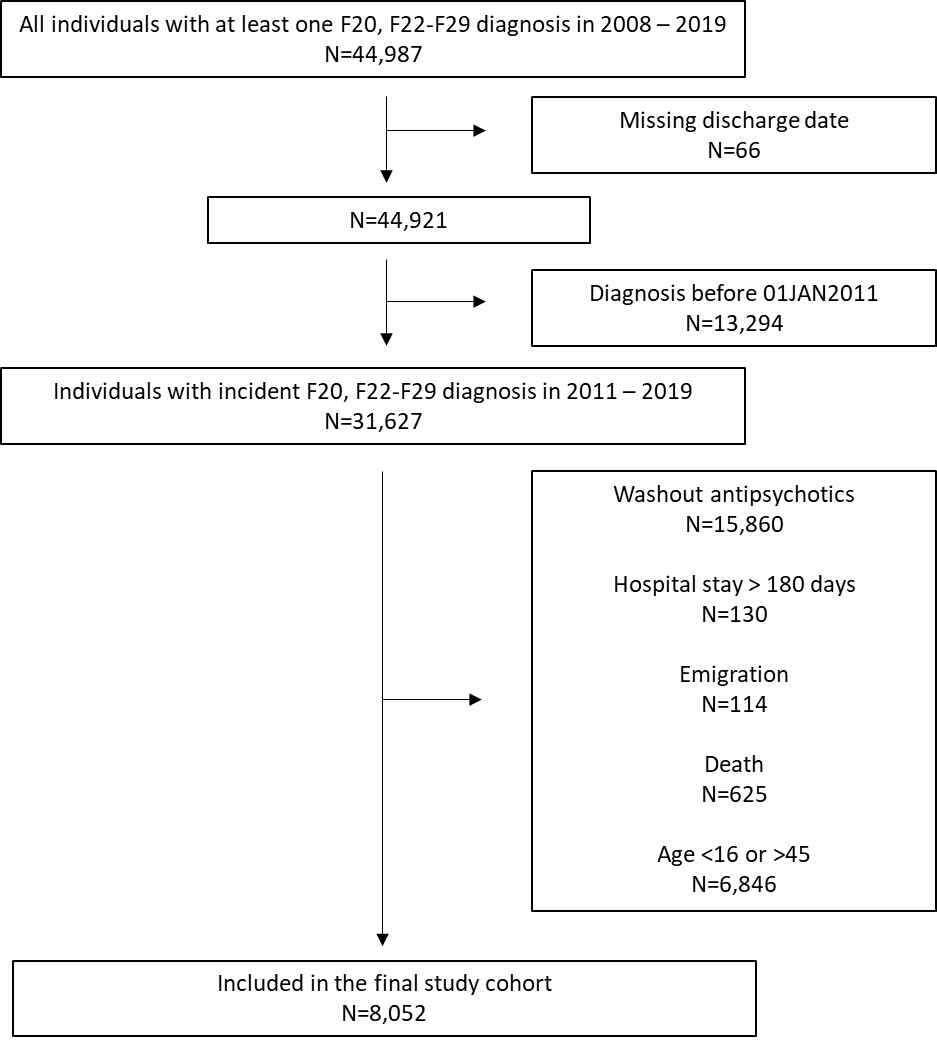
**

**Figure S1.** Flowchart for selection of the study population.

**
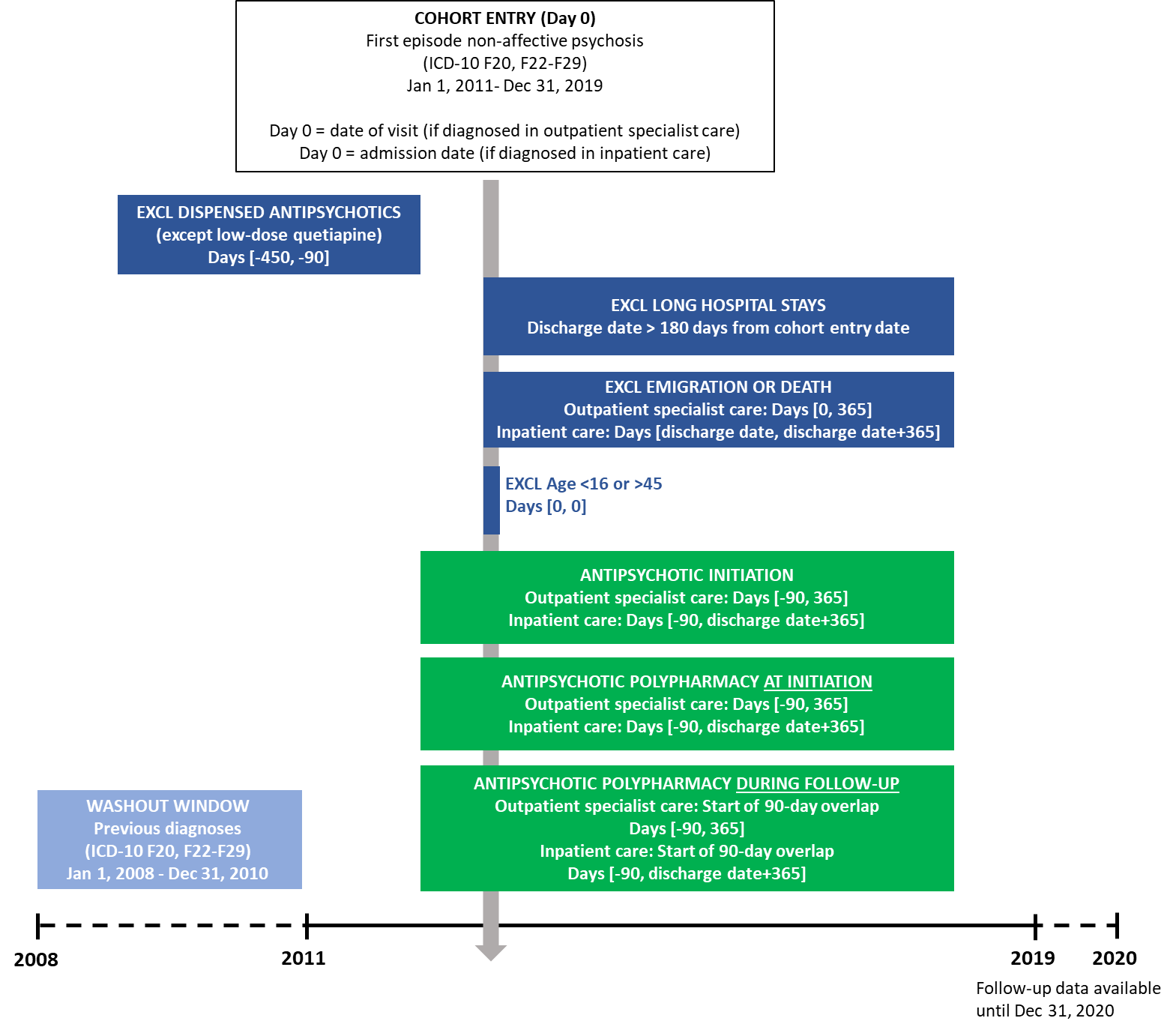
**

**Figure S2.** Illustration of study design and assessment periods. The drug use observation window started from day -90 since diagnoses are derived from specialist health care only. Patients who are first treated in primary care may initiate antipsychotics before a diagnosis is registered in specialist health care.

**Table S1.** ICD-10 codes for psychosis-spectrum disorders included in the study.

| **ICD-10** | **Description** |
| --- | --- |
| F20 | Schizophrenia |
| F22 | Persistent delusional disorders |
| F23 | Acute and transient psychotic disorders |
| F24 | Induced delusional disorder |
| F25 | Schizoaffective disorders |
| F28 | Other nonorganic psychotic disorders |
| F29 | Unspecified nonorganic psychosis |

ICD-10 = International Classification of Diseases, 10th revision

**Table S2.** Definition of covariates and assessment periods.

| **Covariate** | **Coding system** | **Definition/description** | **Assessment period** |
| --- | --- | --- | --- |
| Education | Not applicable | Highest achieved level of education (lower secondary school or less, upper secondary school, tertiary education, no data on education) | The year before cohort entry year |
| Employment | Not applicable | Status on the labour market in a reference week (employed/unemployed) | The year before cohort entry year |
| Disability pension* | Not applicable | At least one period of disability pension (of more than one day) | -1 to -365 days before cohort entry date |
| Living alone | Not applicable | One-person household | The year before cohort entry year |
| Substance use disorder | ICD-10 | F10-F19 (excl. F17) | -1 to -1095 days before cohort entry date |
| Bipolar disorder | ICD-10 | F30, F31 | -1 to -1095 days before cohort entry date |
| Depression | ICD-10 | F32-F34, F38, F39 | -1 to -1095 days before cohort entry date |
| Anxiety disorder | ICD-10 | F40-F42, F44 | -1 to -1095 days before cohort entry date |
| Stress-related disorder | ICD-10 | F43 | -1 to -1095 days before cohort entry date |
| Personality disorder | ICD-10 | F60-F66, F68, F69 | -1 to -1095 days before cohort entry date |
| Hyperkinetic disorders | ICD-10 | F90 | -1 to -1095 days before cohort entry date |
| Anxiolytics | ATC | N05B | -1 to -90 days before cohort entry date (modelled 90-day drug use period ongoing at least 1 day during the time window) |
| Hypnotics and sedatives | ATC | N05C | -1 to -90 days before cohort entry date (modelled 90-day drug use period ongoing at least 1 day during the time window) |
| Antidepressants | ATC | N06A | -1 to -90 days before cohort entry date (modelled 90-day drug use period ongoing at least 1 day during the time window) |

ICD-10 = International Classification of Diseases, 10th revision; ATC = Anatomical Therapeutic Chemical

*Only individuals 18 years and above can receive disability pension.

**Table S3.** Distribution (%) of antipsychotic drugs initiated within the first year after first-episode non-affective psychosis according to cohort entry year during 2011-2019. The denominator is number of individuals initiating antipsychotics the respective years.

|  | **2011** | **2012** | **2013** | **2014** | **2015** | **2016** | **2017** | **2018** | **2019** | **Trend**** |
| --- | --- | --- | --- | --- | --- | --- | --- | --- | --- | --- |
| olanzapine | 33.3 | 29.4 | 34.5 | 37.9 | 32.7 | 35.8 | 37.9 | 36.0 | 34.5 | 0.48 |
| quetiapine | 20.3 | 24.8 | 18.2 | 20.1 | 23.5 | 23.6 | 19.2 | 21.5 | 20.4 | -0.06 |
| aripiprazole | 10.8 | 10.2 | 9.5 | 11.7 | 12.2 | 13.5 | 11.4 | 11.5 | 12.4 | 0.26 |
| AP polypharmacy* | 8.3 | 7.6 | 9.2 | 8.4 | 8.6 | 7.6 | 9.3 | 9.0 | 9.8 | 0.15 |
| other oral AP | 13.6 | 9.6 | 9.2 | 9.4 | 8.4 | 7.2 | 5.5 | 6.5 | 8.1 | **-0.68** |
| risperidone | 8.9 | 8.7 | 9.0 | 7.5 | 7.3 | 5.9 | 8.1 | 7.2 | 7.4 | -0.23 |
| LAIs | 2.8 | 7.3 | 9.5 | 4.4 | 6.0 | 5.7 | 8.1 | 7.6 | 6.4 | 0.23 |
| clozapine | 1.9 | 2.3 | 0.8 | 0.5 | 1.3 | 0.7 | 0.5 | 0.7 | 1.0 | **-0.14** |

AP = antipsychotic; LAIs = long-acting injectables

* Specific drug substances refer to oral antipsychotics and all long-acting injectables are categorized as “LAIs”. AP polypharmacy refers to situations when two or more antipsychotics were dispensed on the same day. Also, initiation with oral and LAI formulation of the same drug substance is categorized as AP polypharmacy.

** Trend (annual change) calculated with linear regression; bolded values indicate p<0.050
